# Contact Endoscopy – Narrow Band Imaging (CE-NBI) data set for laryngeal lesion assessment

**DOI:** 10.1038/s41597-023-02629-7

**Published:** 2023-10-21

**Authors:** Nazila Esmaeili, Nikolaos Davaris, Axel Boese, Alfredo Illanes, Nassir Navab, Michael Friebe, Christoph Arens

**Affiliations:** 1 Department of Otorhinolaryngology, Head and Neck Surgery, Justus Liebig University of Giessen, 35392 Giessen, Germany; 2 Chair for Computer Aided Medical Procedures and Augmented Reality, Technical University of Munich, 85748 Munich, Germany; 3 SURAG Medical GmbH, 04103 Leipzig, Germany; 4 Department of Otorhinolaryngology, Head and Neck Surgery, Giessen University Hospital, 35392 Giessen, Germany; 5 Department of Otorhinolaryngology, Head and Neck Surgery, Magdeburg University Hospital, 39120 Magdeburg, Germany; 6 INKA-Innovation Laboratory for Image Guided Therapy, Medical Faculty, Otto-von-Guericke University Magdeburg, 39120 Magdeburg, Germany; 7 Department of Biocybernetics and Biomedical Engineering, AGH University Kraków, 30-059 Kraków, Poland; 8 CIBE - Center for Innovation, Business Development & Entrepreneurship, FOM University of Applied Sciences, 45141 Essen, Germany

**Keywords:** Cancer imaging, Endoscopy

## Abstract

The endoscopic examination of subepithelial vascular patterns within the vocal fold is crucial for clinicians seeking to distinguish between benign lesions and laryngeal cancer. Among innovative techniques, Contact Endoscopy combined with Narrow Band Imaging (CE-NBI) offers real-time visualization of these vascular structures. Despite the advent of CE-NBI, concerns have arisen regarding the subjective interpretation of its images. As a result, several computer-based solutions have been developed to address this issue. This study introduces the CE-NBI data set, the first publicly accessible data set that features enhanced and magnified visualizations of subepithelial blood vessels within the vocal fold. This data set encompasses 11144 images from 210 adult patients with pathological vocal fold conditions, where CE-NBI images are annotated using three distinct label categories. The data set has proven invaluable for numerous clinical assessments geared toward diagnosing laryngeal cancer using Optical Biopsy. Furthermore, given its versatility for various image analysis tasks, we have devised and implemented diverse image classification scenarios using Machine Learning (ML) approaches to address critical clinical challenges in assessing laryngeal lesions.

## Background & Summary

Artificial intelligence (AI) algorithms have been introduced in the last years to otolaryngology and other disciplines to assist the clinical interpretation of endoscopic image or video data, establishing the new field of videomics^1^. The different approaches include image processing, Machine Learning (ML), and Deep Learning (DL) algorithms based on Convolutional Neural Networks (CNN) and require large data sets of endoscopic images to train and test the AI models. The use of videomics in laryngeal endoscopy is particularly challenging because of the complex anatomy and dynamic nature of the vocal folds and the lack of large data sets of high-definition images^2,3^.

From the clinical point of view, most patients with glottic or vocal fold pathologies are presented to Otolaryngologists due to persistent hoarseness or dysphonia. Hoarseness can be attributed to various causes, including local inflammation, functional voice disorders, and benign, premalignant or malignant laryngeal lesions. The differentiation between these possible diagnoses based on the endoscopic examination of the larynx defines the therapeutic modality. In contrast, the early diagnosis of laryngeal cancer or precancerous lesions is paramount for the patient’s prognosis and preservation of laryngeal function. In cases of suspected malignancy, surgical intervention with biopsy or complete excision is necessary for the histopathological examination of the lesion^4,5^.

The standard method for the clinical evaluation of the vocal folds is laryngoscopy using rigid or flexible scopes in the context of White Light Endoscopy (WLE). This procedure allows the recording of endoscopic videos and images for future reference, teaching or research purposes^4,6^. During laryngoscopy, the evaluation of vascular changes in the vocal folds is crucial for taking clinical diagnostic decisions . Over the years, several classification systems have been proposed for this purpose^7–9^. Recently, image Enhanced Endoscopy (EE) techniques like Narrow Band Imaging (NBI) or Enhanced Contact Endoscopy (ECE) have been introduced and have shown to be clinically helpful in differentiating between benign and malignant laryngeal lesions with higher accuracy compared to WLE^10–12^. By providing higher contrasted vascular patterns, NBI can help overcome the ‘umbrella effect’ caused by vocal fold leukoplakia, a white plaque covering the mucosa that can obscure vascular changes^12,13^. However, the evaluation of vascular patterns largely depends on the classification system used and is subject to the individual experience of each endoscopist^14,15^.

The use of AI can reduce subjectivity in evaluating endoscopic images and provide valuable assistance to Otolaryngologists in making a clinical diagnosis. Currently, most applications focus on differentiating between normal vocal folds and benign or malignant lesions using WLE or NBI laryngoscopy of the vocal folds based on image texture or vasculature analysis^2,3^. The proposed approaches vary considerably in image pre-processing methods and the number of images used to train and test the algorithm. In distinguishing between benign and malignant lesions, a recent meta-analysis by Zurek *et al*. reported a diagnostic sensitivity of 0.80 to 0.95 and a specificity of 0.86 to 0.99, concluding that the diagnostic accuracy of ML-based methods is comparable to that of experienced professionals. Therefore, AI can be a valuable assistant tool for young or inexperienced professionals^16^.

During the last few years, we have tested the combined use of NBI with intraoperative Contact Endoscopy (CE-NBI) for a more detailed examination of highly contrasted vascular patterns and detection of minute vascular changes through high magnification. Enhanced visualization of vascular patterns through CE-NBI allows better differentiation between benign and malignant lesions. Examining the vascularization network on the edge of the lesion is beneficial in the presence of vocal fold leukoplakia. This approach has been tested in the clinical setting, leading to high diagnostic accuracy values and interrater reliability^14,15^. We have further proposed several feature extraction methods combined with ML-based methods for an automated vascular pattern categorization on CE-NBI images^17,18^. Moreover, a DL-based approach was developed based on the Transfer Learning concept to reach a more objective assessment of the laryngeal lesions using CE-NBI images^19^. Given that there are only a few public data sets in the field of laryngeal endoscopy with a limited number of images available^20–22^, in this paper, we aim to introduce the public CE-NBI data set and share the value of this kind of medical data we have collected through the years with the scientific community. The provided laryngeal endoscopy data set contains 11144 labeled images of 210 patients, the most extensive published data set on CE-NBI images. The present data set with three types of labeling is suitable for various clinical investigations and computer-based algorithm developments. The publication of such data could promote multi-center cooperation and further studies on the usefulness of videomics in laryngeal diagnostic and treatment procedures.

## Methods

### Ethical consideration

All patients were required to give informed and written consent to participate in the study. Moreover, all patients’ data were anonymized, and a random name was assigned to each file before it was exported from Magdeburg University Hospital’s central server. The local ethics committee reviewed and approved the study protocol, which meets the criteria of the latest version of the Declaration of Helsinki (report no. 49/18), and the Data Safety Office of Magdeburg University Hospital approved the release of the CE-NBI images under an open license.

### Data acquisition

The CE-NBI video scenes of adult patients with suspicious benign, premalignant, and malignant lesions in the vocal fold have been captured during the endoscopy-based diagnostic procedure before performing micro-laryngoscopy surgery. The procedure was conducted in the Department of Otorhinolaryngology, Head and Neck Surgery in Magdeburg University Hospital, Germany, from 1 January 2015 to 31 December 2021. Following micro-laryngoscopy surgery, the tissue samples collected from the vocal fold were sent for histopathological examination. The final diagnostic result was recorded and was used later to label the data. The video acquisition setup included the Evis Exera II /Evis Exera III Video Systems with a xenon light source plus an integrated NBI filter (Olympus Medical Systems, Hamburg, Germany) as well as a rigid 30-degree contact endoscope (Karl Storz, Tuttlingen, Germany). The magnification degree of the endoscopic system was adjusted in combination with 60x or 150x magnification of the contact endoscope to reach the optimum visualization of the vascular patterns during the procedure.

All the video scenes were acquired before performing an excisional biopsy or cordectomy of the vocal fold in Audio Video Interleave (AVI) format with 30 frames per second (fps).

### Data preparation

The CE-NBI images were extracted and selected from the captured video scenes in a three-step process. For each video, an experienced person working in the field of medical video and image processing (Ph.D. student) manually selected the time intervals where the video’s quality was good and acceptable to see the blood vessels (good resolution without blur and artifacts). Then, one in every three frames was extracted from the selected intervals in the videos using an automatic algorithm in MATLAB R2019a and MATLAB R2020a. In the last step, two experienced Otolaryngologists in joint sessions visually evaluated the series of extracted CE-NBI images per patient and selected images with unique and non-redundant vascular patterns in the mucosa of the target lesion for the data set.

The data annotations were followed in two paths, resulting in three different labels per image. First, the histopathology results from the surgical biopsy were used to label the images of each patient to:

- The diagnosed laryngeal histopathology label (multi-class labeling).
- The lesion-type benign-malignant label (binary labeling).
  Second, the macroscopic appearance of the vocal fold, independent of the histopathology results, was assessed visually by two experienced Otolaryngologists to generate:
- The leukoplakia diagnosis label, according to the presence or absence of the vocal fold leukoplakia (binary labeling).

**Fig. 1:**
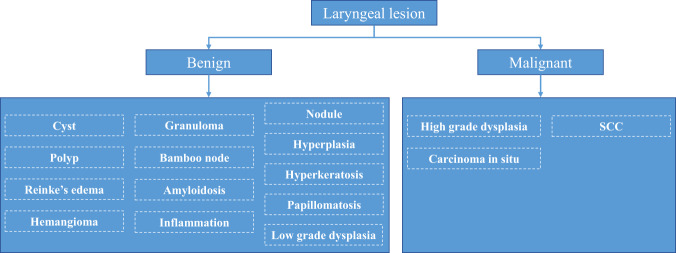
Laryngeal histopathologies.

In total, the laryngeal histopathologies were divided into 16 different categories. As it is presented in Fig. 1 and according to World Health Organization (WHO) classification, the benign lesions included histopathologies such as cyst, polyp, reinke’s edema, bamboo nodes, nodule, granuloma, amyloidosis, inflammation, hemangioma, papillomatosis, hyperplasia, hyperkeratosis. Regarding the premalignant lesions, low grade dysplasia was labeled benign because of its low malignant transformation potential, while high grade dysplasia and carcinoma *in situ* were labeled malignant. Hence, the malignant lesions contained histopathologies as high grade dysplasia, carcinoma *in situ*, and squamous cell carcinoma (SCC).

## Data Records

**Fig. 2:**
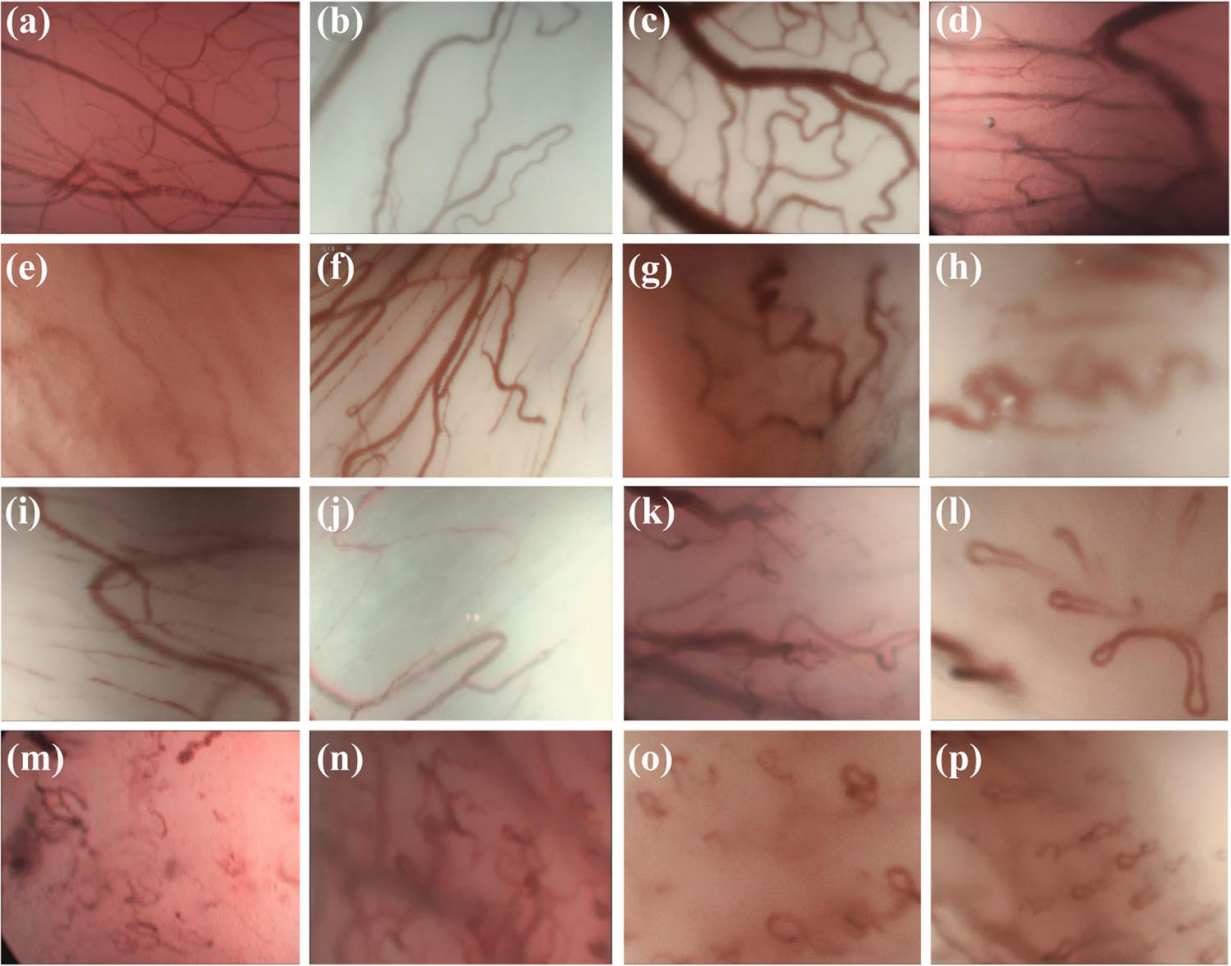
The example of CE-NBI images for every histopathology. (**a**) Cyst, (**b**) Polyp, (**c**) Reinke’s edema, (**d**) Hemangioma (**e**) Granuloma, (**f**) Bamboo nodes, (**g**) Amyloidosis, (**h**) Inflammation, (**i**) Nodule, (**j**) Hyperplasia, (**k**) Hyperkeratosis, (**l**) Papillomatosis, (**m**) Low grade dysplasia, (**n**) High grade dysplasia, (**o**) Carcinoma *in situ*, and (**p**) SCC.

The full data set, including all images and labels, is available from Zenodo Repository (https://zenodo.org/) and can be downloaded from^23^. In total, the data set consists of two main data categories: benign images and malignant images. In each category, the images of every patient are ordered according to the laryngeal histopathology classes. In this way, the images belonging to each class are stored in the corresponding folder of the category it belongs to. For example, the cyst folder in the benign category includes total CE-NBI images of all patients diagnosed with a cyst or the SCC folder in the malignant category contains all CE-NBI images of patients with SCC. Figure 2 shows one example of CE-NBI images for every histopathology. Moreover, one general Excel file is provided to map the image files of each patient to image labels (diagnosed laryngeal histopathology, lesion-type benign-malignant, and leukoplakia diagnosis) and image dimension. The data set has a total size of 1.34 GB, 945 MB for benign images and 422 MB for malignant images.

The CE-NBI data set contains 11144 labeled images of 210 patients with a resolution of 96 dpi stored in JPEG (Joint Photographic Experts Group) format that can have different dimensions as specified in Table 1.

**Table 1:** Image dimensions in CE-NBI data set.

Image dimension (in pixels)	Number of images
1280 × 1008	6701
1736 × 1080	1266
1842 × 1080	548
720 × 544	1153
868 × 540	1476

**Fig. 3:**
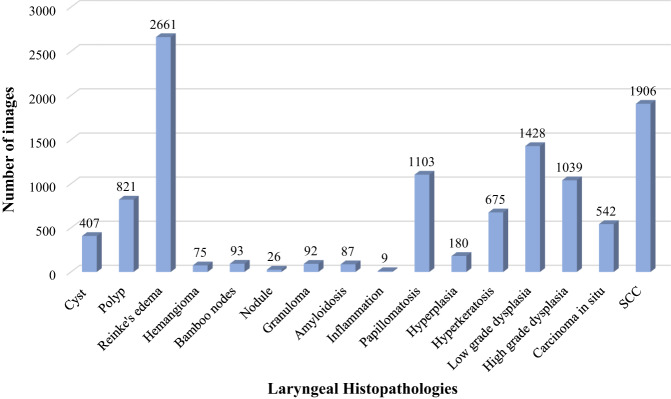
Number of CE-NBI images in the data set per laryngeal histopathology.

**Fig. 4:**
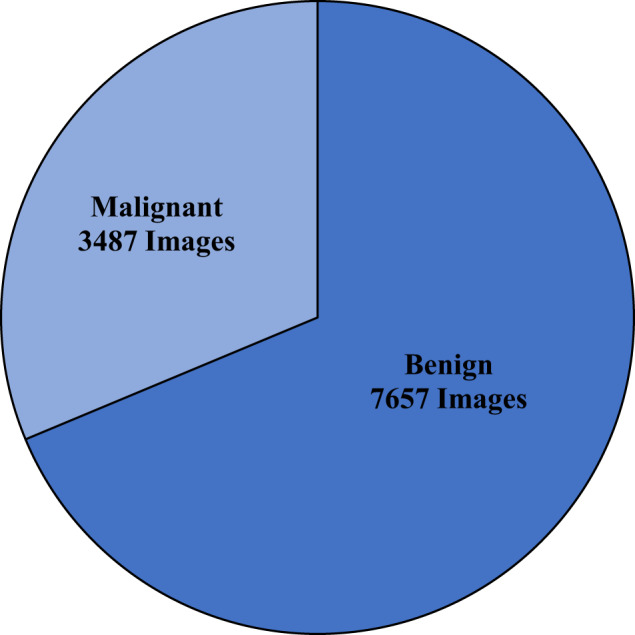
Number of CE-NBI images in the data set per type of laryngeal lesion.

Table 2 represents an overview of the total number of CE-NBI images and patients based on the type of laryngeal lesions and histopathologies. Additionally, a more detailed visualization of the number of images per 16 different histopathology classes and two lesion-type classes is presented in Figs. 3, 4. The benign class with 7657 CE-NBI images of 150 patients from 13 different histopathologies contains 69% of total data. In this class, reinke’s edema with 2661 images of 45 patients has the highest number of images among other histopathologies. The remaining 31% of the total data belongs to the malignant class with 3487 CE-NBI images of 60 patients with 3 different histopathologies. SCC with 1906 images of 30 patients has the highest number of images among other malignant histopathologies.

**Table 2:** Total number of CE-NBI images and patients in the data set according to the type of laryngeal lesion and histopathology.

Type of lesion	Number of patients per lesion	Number of images per lesion	Histopathology	Number of patients per histopathology	Number of images per histopathology
Benign	150	7657	Cyst	9	407
			Polyp	20	821
			Reinke’s edema	45	2661
			Hemangioma	2	75
			Bamboo nodes	1	93
			Nodule	1	26
			Granuloma	3	92
			Amyloidosis	3	87
			Inflammation	1	9
			Papillomatosis	22	1103
			Hyperplasia	5	180
			Hyperkeratosis	14	675
			Low grade dysplasia	24	1428
Malignant	60	3487	High grade dysplasia	18	1039
			Carcinoma *in situ*	12	542
			SCC	30	1906

**Fig. 5:**
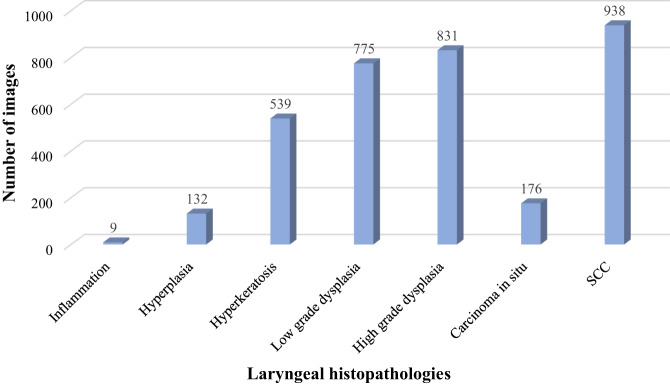
Number of CE-NBI images of the leukoplakia group in the data set based on the laryngeal histopathology.

As was mentioned before, all images have the leukoplakia diagnosis label. Leukoplakia can be associated with a broad spectrum of histopathological diagnoses, from hyperplasia to malignant transformation. From all the images in the CE-NBI data set, 3400 images of 65 patients belong to leukoplakia cases. Table 3 summarizes the number of images and patients with leukoplakia per type of laryngeal lesion and histopathology. As is shown in Fig. 5, low grade dysplasia with 775 and SCC with 938 images have the highest number of CE-NBI images in benign and malignant categories for leukoplakia cases, respectively.

**Table 3:** Total number of CE-NBI images and patients with leukoplakia, classified based on the type of laryngeal lesion and histopathology.

Type of lesion	Number of patients per lesion	Number of images per lesion	Histopathology	Number of patients per histopathology	Number of images per histopathology
Benign	28	1455	Inflammation	1	9
			Hyperplasia	3	132
			Hyperkeratosis	10	539
			Low grade dysplasia	14	775
Malignant	37	1945	High grade dysplasia	14	831
			Carcinoma *in situ*	7	176
			SCC	16	938

## Technical Validation

Several studies were conducted to provide a better vision and understanding of the technical aspects of the CE-NBI data set. While the data set is well suited for various image processing and ML-based tasks, we have implemented multiple image classification scenarios on parts of this data set. The proposed approaches addressed the main clinical issues in the area of laryngeal cancer diagnosis and performed well on CE-NBI image classification based on the type of laryngeal lesion and histopathology. The value of each study is briefly explained below, along with their results as summarized in Table 4.

**Table 4:** Summary of the technical validation results on CE-NBI data set.

Method	Data	Classification	Accuracy	Sensitivity	Specificity
GF + Support Vector Machines^17^	32 patients, 1485 images	Vascular patterns	0.973	0.980	0.983
GF + k-Nearest Neighbors^17^	31 patients, 1355 images	Benign-malignant lesion	0.912	0.871	0.933
GF + Random Forests^17^	20 patients, 890 images	Benign histopathology	0.906	0.900	0.965
GF + Random Forests^17^	11 patients, 465 images	Malignant histopathology	0.884	0.879	0.943
CyEfF + Support Vector Machines^18^	48 patients, 2701 images	Benign-malignant lesion	0.875	0.826	0.920
CyEfF + GF + k-Nearest Neighbors^18^	48 patients, 2701 images	Benign-malignant lesion	0.966	0.959	0.973
CyEfF + GF + k-Nearest Neighbors^27^	40 patients, 1998 images	Leukoplakia cases, Benign-malignant lesion	0.917	1	0.897
Pre-trained ResNet50^19^	146 patients, 8181 images	Benign-malignant lesion	0.835	NA	NA
Pre-trained DenseNet121	210 patients, 11144 images	Benign-malignant lesion	0.876	0.820	0.901
Pre-trained EfficientNetB0V2	210 patients, 11144 images	Benign-malignant lesion	0.878	0.822	0.902
Pre-trained ResNet50V2	210 patients, 11144 images	Benign-malignant lesion	0.907	0.846	0.934
Ensemble model of DenseNet121, EfficientNetB0V2, ResNet50V2	210 patients, 11144 images	Benign-malignant lesion	0.928	0.877	0.951

In the first study, we used a set of hand-crafted features in combination with supervised ML classifiers to evaluate the geometrical characteristics of vascular patterns in CE-NBI images. The proposed approach confirmed the relevance of the vascular structures to the laryngeal histopathology as well as to the type of laryngeal lesion^17,24^. This evaluation was continued by performing a classification scenario similar to the routine clinical procedure, where the performance of the proposed approach was compared to the diagnosis decision of Otolaryngologists regarding the type of laryngeal lesion based on vascular patterns in CE-NBI images^25,26^. The presented results showed the efficiency of a computer-based solution to assist Otolaryngologists when there are disagreements regarding the final diagnosis.

In the next round of analyzing CE-NBI images, the textural aspects of these images were studied using a novel set of features called Cyclist Effort Features (CyEfF)^18^. The presented results demonstrated the importance of textural characteristics in CE-NBI images because the CyEfF, in combination with the previously discussed Geometrical Features (GF), could improve the classification performance of ML classifiers. Additionally, the combination of these two sets of hand-crafted features showed high performance in the supervised classification of CE-NBI images of leukoplakia cases according to the type of laryngeal lesion^27^.

In another evaluation that included around 73% of the current data set, a pre-trained Residual Networks (ResNet50) architecture^28^ was combined with the cut-off layer technique as a fully automatic approach for the CE-NBI image classification based on benign and malignant lesions^19^. The given results could prove the significance of labeled data in the ML-based image classification task. Besides, the proposed model could be a solution for the subjective assessment of laryngeal lesions in clinical settings.

**Fig. 6:**
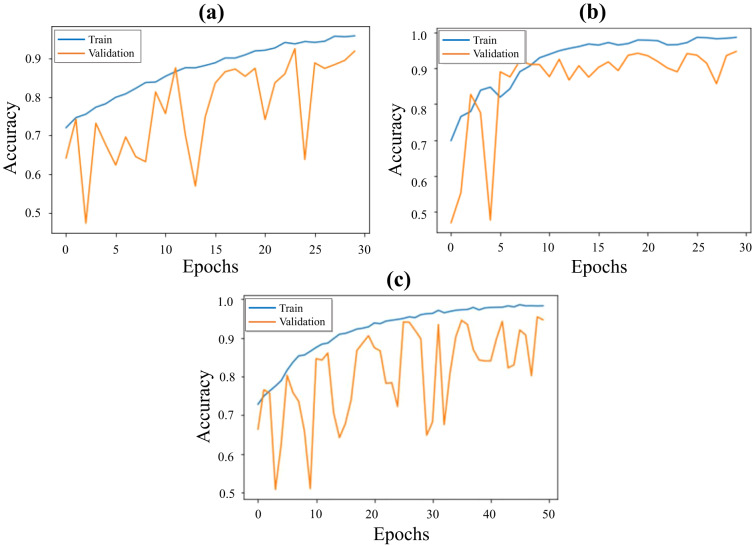
Training and validation results of three pre-trained models on the CE-NBI image data set. Accuracy graph of final models: (**a**) DenseNet121, (**b**) EfficientNetB0V2, and (**c**) ResNet50V2.

**Fig. 7:**
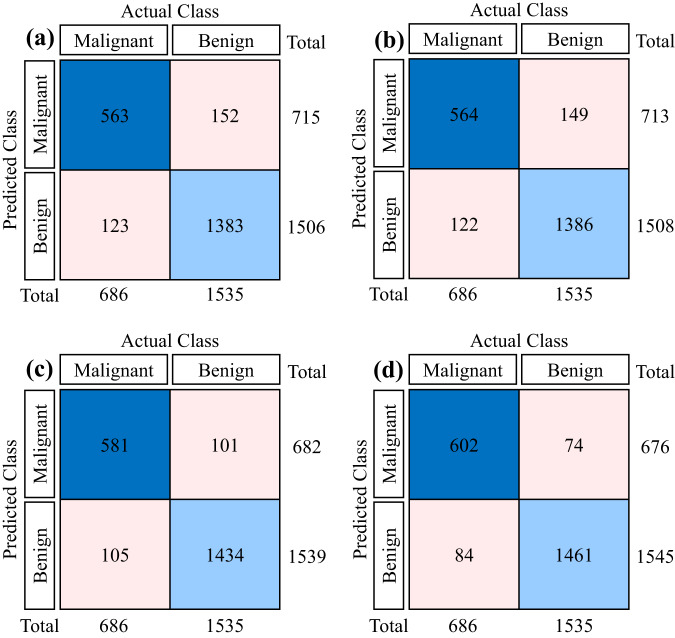
Testing results of three pre-trained models and the ensemble model on the CE-NBI image data set. Confusion matrix: (**a**) DenseNet121, (**b**) EfficientNetB0V2, (**c**) ResNet50V2, and (**d**) Ensemble model.

In the current study, we have developed a new DL-based approach using the ensemble modeling technique to combine the power of different architectures for CE-NBI image classification. We have validated and tested this approach on the entire publicly available CE-NBI data set to perform an automatic image classification based on benign and malignant classes. First, we have used the Transfer Learning concept and selected three pre-trained architectures, including Dense Convolutional Neural Network (DenseNet121)^29^, Efficient Network (EfficientNetB0V2)^30^, and Residual Networks (ResNet50V2) for this classification task. We have applied fine-tuning strategy on every architecture to reach the optimum performance, speed up the training, and overcome the problem with the small data set size. The final parameters after fine-tuning for each architecture were set as follows: the batch size of 32, the number of epochs equals 30 to 50, the input shape of the image as 224 × 224 pixels, and early stopping with the patience of 5 epochs. After training each fine-tuned architecture, an ensemble model was constructed using parallel topology, and predictions were made using model averaging. Model averaging is a well-known method of ensemble learning in which ensemble prediction is calculated as an average of the number of predictions, and each model makes an equal contribution to the final prediction. For the evaluation, we employed the hold-out approach to split the data into 80% training and 20% testing. In this way, we ensured that there was no overlap between the two sets and that images of every patient were just conjoined to separate groups. Furthermore, we have applied image augmentation techniques on the training set, including geometric transformations such as vertical and horizontal flips, to solve the issue of unbalanced data between two classes and avoid the possible occurrence of over-fitting. After this process, the number of images in the training set was increased to 11,680 data, with 6080 benign and 5600 malignant images. We have trained and validated three fine-tuned DenseNet121, EfficientNetB0V2, and ResNet50V2 on the training set where Fig. 6 shows the accuracy track for each model. Then, we tested each fine-tuned model along with the final ensemble model on the unseen data in the testing set, where Fig. 7 represents the confusion matrix of this experiment for each model. As presented in Table 4, the proposed ensemble model indicated a higher performance than the other approaches developed based on the Transfer Learning concept in CE-NBI image classification, showing the significance of the data in developing DL-based methods and solving complex image classification tasks.

It is important to highlight that we mainly explored the CE-NBI image data set for classification tasks based on laryngeal lesion type (benign versus malignant classes). Therefore, further technical research studies could be beneficial to explore the CE-NBI data set for multi-class image classification scenarios based on diagnosed laryngeal histopathology.

## Usage Notes

The presented data set is a worthwhile source of data with 16 different diagnoses of various patients that can play a crucial role in the future advancement of laryngeal lesion diagnosis and treatment. The CE-NBI data set is generated from the data acquired in the existing clinical setting. Therefore, it has some limitations for clinical and technical research applications that are outlined as follows:

As the data acquisition strategy was focused on patients with suspicious benign, premalignant, and malignant lesions, the data set does not include a control group with healthy cases. Therefore, the data set can mainly be used in diagnostic studies, such as differentiation between benign and malignant pathologies, and it is not a suitable source for studies focusing on detecting laryngeal tissue changes.

The distribution of CE-NBI images of various histopathologies in the data set represents their numerical occurrence in the actual clinical setting. Nevertheless, it causes an imbalanced distribution of data among different classes. In the given data set, there are nearly double the number of images in the benign category compared to the malignant group. This issue may pose challenges in the development of ML-based algorithms. For example:

- the algorithms could tend to be biased towards the majority class because they have more data to learn from,
- the trained models could generalize poorly to new and unseen data because the minority class is not well-represented in the training data,
- and the final models could overfit the training data to correctly classify the minority class because they have learned non-valuable patterns from the images in the training set.

The problem of imbalanced data is a common issue in clinical and technical studies that can be addressed with data resampling. The clinicians usually study 5 to 10 images per patient to arrive at the diagnosis decision. Therefore, the undersampling of the patient images in the majority class could be a potential solution to the imbalanced data issue. On the other hand, the development of ML-based approaches benefits from the considerable amount of data. Thus, the oversampling of data in minority groups using different techniques, such as data augmentation, could deal with this problem. Moreover, designing cost-sensitive learning algorithms and applying ensemble modeling techniques could be other solutions to improve the performance of ML-based approaches on the imbalanced CE-NBI image data set.

The small number of occurrences for certain histopathologies in the existent clinical setting has resulted in a limited number of CE-NBI images for these cases in the data set. Some classes, such as inflammation, nodule, amyloidosis, granuloma, bamboo nodes, and hemangioma, contain less than 100 CE-NBI images which may not be sufficient for the DL-based model to learn the underlying patterns of the data. This issue could hinder the development of ML-based methods for multi-class classification tasks based on diagnosed laryngeal histopathology. However, conducting a multi-class classification scenario with three classes, including benign, premalignant, and malignant groups, could be the next step toward histopathological diagnosis.

The application of CE-NBI images for laryngeal lesion assessment is limited to the research area where the data has to be acquired intraoperatively and under general anesthesia. In this condition, there is no standard and defined protocol for data acquisition. We could handle this issue by creating a study protocol in Magdeburg University Hospital to perform the data collection task. However, the lack of standard workflow in this step could result in data variability among different clinical centers because each hospital can follow an individual workflow where the characteristics of the images, such as magnification and resolution, could differ from the other centers. Future studies could overcome this limitation by proposing a universal data acquisition protocol for CE-NBI application or focusing on data collection for examination methods that are part of the routine clinical procedure, such as flexible endoscopy.

## Data Availability

The CE-NBI image data set can be used without any other code for technical and clinical image classification and assessment purposes. However, the algorithms already developed on this data set are available (https://github.com/NazilaEsmaeili/CE-NBI-Classification) to compare the performance of the newly developed approaches with the existing methods. The provided codes are available for public access only for research purposes.
